# QEEG-Guided rTMS in Pediatric ASD with Contextual Evidence on Home-Based tDCS: Within-Cohort Reanalysis and Narrative Contextualization

**DOI:** 10.3390/children12111453

**Published:** 2025-10-25

**Authors:** Alptekin Aydin, Ali Yildirim, Ece Damla Duman

**Affiliations:** 1 Department of Clinical Psychology, Health and Life Science Faculty, Atlantic International University, Honolulu, HI 96813, USA; 2 Deepsynaps, Oxford OX4 4GP, UK; ali.yildirim@deepsynaps.com; 3 Cosmos Healthcare, London WC2H 9JQ, UK

**Keywords:** Autism Spectrum Disorder, quantitative EEG, repetitive transcranial magnetic stimulation, transcranial direct current stimulation, dorsolateral prefrontal cortex, neuromodulation, pediatric neuropsychiatry, stepped-care model

## Abstract

**Background:** Autism Spectrum Disorder (ASD) affects ~1 in 36 children and is increasingly studied as a candidate for non-invasive neuromodulation. Two of the most widely applied modalities are quantitative EEG (QEEG)-guided repetitive transcranial magnetic stimulation (rTMS) and transcranial direct current stimulation (tDCS), both targeting the dorsolateral prefrontal cortex (DLPFC). While both have shown promise, questions remain regarding their relative clinical profiles and scalability. **Objective:** To conduct a within-cohort reanalysis of QEEG-guided rTMS outcomes in paediatric ASD and to contextualise these findings alongside published reports of home-supervised tDCS. **Methods:** Individual participant data (*n* = 56, ages 6–17) from a prospective rTMS cohort were reanalysed, focusing on the Social Responsiveness Scale (SRS-2), Autism Diagnostic Observation Schedule (ADOS-2), Aberrant Behavior Checklist (ABC), Repetitive Behavior Scale–Revised (RBS-R), and QEEG biomarkers. Findings were then situated within a narrative synthesis of published paediatric tDCS trials, which consistently report caregiver-supervised feasibility but did not provide raw, baseline-adjusted data suitable for reanalysis. **Results:** rTMS was associated with large within-cohort improvements (Hedges’ g ≈ 1.0–1.6), including an 11-point reduction in SRS-2 T-scores, a 12-point reduction in ABC totals, and robust QEEG normalisation (β/γ suppression, α enhancement). Published tDCS studies report moderate, clinically meaningful improvements in social communication, executive functioning, and regulation (Cohen’s d ≈ 0.4–0.6), with excellent adherence and no serious adverse events. **Conclusions:** rTMS produced robust behavioural and neurophysiological improvements within its cohort, while published tDCS trials demonstrate moderate, feasible benefits in home settings. Because of incomplete baseline data and protocol differences, no direct statistical comparison was possible. These findings suggest complementary roles: rTMS as a high-intensity clinic-based intervention, and tDCS as a scalable, family-centred option. A stepped-care framework that combines both modalities should be considered hypothesis-generating only and requires validation in harmonised, randomised controlled trials.

## 1. Introduction

Autism Spectrum Disorder (ASD) is a heterogeneous neurodevelopmental condition affecting approximately 1 in 36 children aged eight years in the United States, with a male:female ratio of nearly 4:1 [1]. Research into ASD outcomes has increasingly focused on identifying neurobiological and behavioural predictors that can guide early intervention. Mews et al. [2] emphasize the value of multimodal markers, including electrophysiological and behavioral data, for understanding variability in ASD presentation and outcomes. Similarly, Moxon-Emre et al. [3] report that repetitive transcranial magnetic stimulation (rTMS) targeting glutamate/glutamine imbalances in the DLPFC may improve social cognition and executive function. Non-invasive neuromodulation techniques such as tDCS have shown promise in enhancing social-cognitive performance. Pereira et al. [4] demonstrated improved outcomes following multichannel anodal tDCS over DLPFC. In parallel, Sandran et al. [5] highlighted the practicality of in-home neuromodulation protocols, offering scalable options for clinical use. Pharmacological and experimental therapies are also under investigation. The CASCADE study by Sannar et al. [6] explores the use of cannabidiol in children with ASD, targeting irritability and behavioral dysregulation. Complementing this, Scheerer et al. [7] examined how sensory phenotypes affect ASD subgroups and may influence treatment responsiveness. Microbiome-based interventions have gained attention as well. Schmitt et al. [8] tested a novel probiotic (SB-121) and reported favourable effects on social behavior. Meanwhile, auditory processing deficits were reaffirmed by Seymour et al. [9], who found reduced auditory steady-state responses in ASD, reflecting broader sensory processing challenges.

Neuromodulatory brain stimulation in pediatric populations remains a key area of innovation. Splittgerber et al. [10] found that multichannel tDCS over left DLPFC was feasible and safe in children. Likewise, van Steenburgh et al. [11] showed that bifrontal tDCS enhanced working memory in high-functioning autistic adults. In terms of biomarkers and diagnostic strategies, Sun et al. [12] combined EEG with eye tracking to identify novel markers of restricted interest. Thoen et al. [13] proposed respiratory-sinus-arrhythmia biofeedback to reduce physiological stress in adolescents with ASD. Combining neuromodulation with cognitive training, Westwood et al. [14] demonstrated that tDCS enhanced cognitive outcomes in boys with ADHD, with translational potential for ASD. Wu et al. [15] further revealed distinct and sometimes conflicting impacts of tDCS versus tRNS on connectivity and memory performance. Neuroimaging studies continue to uncover large-scale disruptions in brain networks associated with ASD. Zeng et al. [16] reported widespread abnormal functional connectivity in children with ASD. Finally, Zheng et al. [17] evaluated transdiagnostic effects of tDCS in treating anxiety and depression, providing insight into potential co-treatment of emotional dysregulation in ASD.Core characteristics include persistent difficulties in social communication and interaction across contexts, alongside restricted and repetitive behaviours, as set out in the DSM-5-TR [18]. Longitudinal studies show that social-communication impairments are among the strongest predictors of functional outcomes into adolescence and adulthood, often exceeding the prognostic value of global IQ [19,20]. Converging neuroimaging and electrophysiology implicate the frontal components of the “social brain” network—especially the dorsolateral prefrontal cortex (DLPFC)—in ASD [21,22]. Quantitative EEG (QEEG) often reveals elevated β/γ power with reduced posterior α coherence and atypical frontolateral asymmetry, patterns that correlate with social-executive challenges [23]. Such QEEG phenotypes and the value of QEEG for characterizing the autistic brain have been documented extensively [24]

These observations motivate DLPFC-targeted neuromodulation strategies aimed at restoring excitation–inhibition balance and improving top-down regulatory control [25].

Among non-invasive brain stimulation (NIBS) modalities, repetitive transcranial magnetic stimulation (rTMS) and transcranial direct current stimulation (tDCS) have accumulated the most supportive evidence in paediatric ASD [25,26,27,28]. Frequency-specific rTMS can inhibit hyperactive right DLPFC at ≤1 Hz and excite hypoactive left DLPFC at ≥10 Hz, inducing long-term synaptic plasticity that maps onto symptom change [26,28]. tDCS applies low-intensity, polarity-dependent current—most commonly anodal over left DLPFC—to shift cortical excitability and augment learning, with the practical advantage of caregiver-supervised home delivery [27,29,30]. Despite promising results for both modalities, implementation research questions remain: for whom, when, and how should each technology be deployed; what are the realistic effects in routine care; and how might biomarker-guided personalisation improve outcomes [25]. The present work addresses part of this agenda by reporting a within-cohort reanalysis of QEEG-guided rTMS and by contextualising these findings with a narrative summary of home-based tDCS outcomes from the peer-reviewed paediatric literature [29,30]. Crucially, because raw participant-level baseline data were unavailable for the tDCS cohort and because protocols differ (clinic-based 40 sessions vs. home-based 20 sessions), we do not present a direct, quantitative comparison [28].

Objectives. (i) Quantify behavioural and neurophysiological change after QEEG-guided rTMS; (ii) summarise the typical magnitude and feasibility of home-based tDCS from published sources; and (iii) delineate implications for stepped-care designs to be tested prospectively [25,28]. This approach aims to bridge the gap between high-intensity, clinic-based interventions and scalable, family-centered options, potentially leading to more personalized and effective treatments for ASD [31]. This study aims to provide a nuanced perspective on the distinct roles of these neuromodulation techniques in addressing the complex neuropathology of ASD, specifically focusing on the restoration of excitation/inhibition balance within critical neural circuits [32,33]. Among these, quantitative electroencephalography-guided repetitive transcranial magnetic stimulation (rTMS) and transcranial direct current stimulation (tDCS) have emerged as prominent modalities, particularly targeting the dorsolateral prefrontal cortex [31,33]. While both have demonstrated therapeutic promise in addressing the atypical behavioral and cognitive patterns observed in ASD, their relative clinical profiles and scalability in pediatric populations warrant further investigation [31].

This study aims to address this gap by conducting a rigorous reanalysis of QEEG-guided rTMS outcomes within a paediatric ASD cohort, juxtaposing these findings with a comprehensive narrative synthesis of published home-supervised tDCS reports to provide a nuanced understanding of their comparative utility [27,34]. With clinically significant improvements reported across various domains [32], and moderate effect sizes [33], the promising but varied outcomes across both rTMS and tDCS interventions highlight the heterogeneity of ASD itself, implying that individualized treatment approaches may yield superior results given the diverse neuropathological underpinnings [32,35,36]. The individualized alpha frequency has been identified as a critical biomarker for tailoring rTMS treatments, particularly given the frequent comorbidity of sleep disturbances in pediatric ASD [32]. This biomarker can also inform treatment strategies for other common comorbidities in ASD, such as attention deficits and anxiety, by precisely targeting neural oscillations implicated in these symptoms [32]. Furthermore, advancements in machine learning are enabling more precise identification of unique subgroups within ASD based on EEG features, which can further refine personalized neuromodulation strategies [37]. Such individualized approaches are crucial given the varied etiology and clinical presentations of ASD, necessitating interventions that can adapt to each patient’s unique neurophysiological profile to maximize therapeutic efficacy [32,38].

This tailored approach ensures that stimulation parameters, such as pulse frequency and intensity, are optimized for each individual, thereby enhancing treatment efficacy and safety while potentially mitigating side effects [38]. Moreover, recent research highlights the importance of the medial prefrontal cortex in self-agency, suggesting rTMS modulation of this region could serve as a novel biomarker for treatment response [39]. However, the implementation of such advanced, individualized protocols necessitates further investigation into optimal stimulation parameters and long-term efficacy [32]. Ongoing investigations, such as those by the Autism Biomarkers Consortium for Clinical Trials, are critical for identifying reliable biomarkers that can predict treatment response to rTMS and provide more sensitive metrics of neural system engagement [38]. Despite this progress, methodological challenges remain, including the need for larger, more homogeneous samples and standardized outcome measures to adequately capture the subtle yet significant improvements observed in clinical trials [33,40]. Additionally, heterogeneity in ASD presentation necessitates recruitment strategies that consider specific characteristics rather than a blanket ASD diagnosis to enhance the precision and generalizability of rTMS studies [40]. Moreover, the impact of comorbidities such as anxiety and ADHD on resting EEG patterns and treatment outcomes warrants careful consideration in future biomarker research for ASD [40]. This underscores the necessity of moving beyond broad diagnostic categories towards stratified approaches that consider individual neurophysiological profiles and comorbid conditions when designing and evaluating neuromodulation interventions for ASD [34]. These considerations are crucial for advancing the field towards more targeted and effective interventions that can significantly improve the quality of life for individuals with ASD [34,41]

## 2. Study Design

This investigation consisted of two complementary components. First, we performed a within-cohort reanalysis of a paediatric sample that underwent QEEG-guided repetitive transcranial magnetic stimulation (rTMS). Raw participant-level behavioural and neurophysiological data were available, enabling detailed statistical characterisation of treatment effects. Second, we conducted a narrative contextualisation of published paediatric studies applying transcranial direct current stimulation (tDCS) at home under caregiver supervision. Because complete baseline distributions and participant-level data were unavailable for the tDCS cohorts, no direct statistical comparison was possible. We therefore reframed tDCS findings as contextual evidence only, extracted from peer-reviewed literature. This approach allowed for a robust evaluation of rTMS efficacy within a controlled cohort while providing a broader understanding of tDCS applicability in real-world, home-based settings. This dual methodology facilitates an assessment of comparative utility and scalability of both modalities, acknowledging the inherent differences in data availability and study designs. The reanalysis of the rTMS cohort utilized a comprehensive set of outcome measures, including the Social Responsiveness Scale, Autism Diagnostic Observation Schedule, Aberrant Behavior Checklist, Repetitive Behavior Scale–Revised, and quantitative EEG biomarkers, which collectively provide a multi-faceted evaluation of treatment efficacy [32].

### 2.1. Participants (rTMS Cohort)

Fifty-six children and adolescents (ages 6–17 years, mean 9.4 ± 2.8; 71% male) with DSM-5 and ADOS-2–confirmed ASD were included. Participants were recruited via outpatient neurology clinics, neurodevelopmental centres, and specialised education schools between 2021 and 2023. Inclusion criteria: confirmed diagnosis of ASD; stable psychotropic medication for at least four weeks; caregiver ability to support 40-session protocol; normal baseline neurological exam; QEEG screening demonstrating targetable prefrontal abnormalities without epileptiform discharges. Exclusion criteria included a history of seizures, severe head trauma, metal implants, or any other contraindication for rTMS or QEEG. The cohort comprised individuals aged 6–17 years, encompassing a spectrum of ASD severity to enhance the generalizability of findings [32]. Exclusion criteria: history of seizures or active epilepsy; cranial implants or metallic devices; severe intellectual disability precluding protocol compliance; unstable psychiatric or systemic medical conditions; concurrent participation in other interventional trials. The mean age of participants was 11.5 years (SD = 2.8, range 6–17), with a male-to-female ratio of approximately 4:1, reflecting the known epidemiological prevalence of ASD. Ethical considerations: All procedures were approved by the Atlantic International University Ethics Committee and adhered to international paediatric NIBS safety guidelines [27]. Written informed consent was obtained from caregivers, and age-appropriate assent was obtained from participants when feasible. The study protocol, including all methods and materials, was developed and implemented in accordance with the Declaration of Helsinki. Parents of participants also provided informed consent, and patients, where possible, signed the informed consent form approved by the Ethics Committee, underscoring the commitment to ethical research practices [33].

### 2.2. QEEG Acquisition and Target Identification

Resting-state 21-channel EEG was acquired in eyes-open and eyes-closed conditions, band-pass filtered (0.5–70 Hz), and artefact-corrected. Spectral analysis was conducted with fast Fourier transform, yielding absolute and relative power values across δ, θ, α, β, and γ bands. Data were converted into age-normed z-scores against a normative database. Prefrontal hyperactivity (β/γ elevation) or α deficits over DLPFC guided target selection (F3/F4). Individualised montages were registered to cranial landmarks; neuronavigation was used when available. This individualized approach ensured precise stimulation delivery to areas of identified neurophysiological dysregulation. Specifically, alpha frequency targeting was informed by identifying the dominant peak frequency within the 8–13 Hz range and multiplying it by the nearest higher harmonic frequency of the ECG [32]. Furthermore, coherence analysis was employed to assess functional connectivity anomalies within and between brain regions, providing additional insights for targeting [42].

The mean individual alpha frequency was determined as a key biomarker for guiding rTMS, exhibiting a significant shift post-treatment [32]. For instance, significant improvements in Individual Alpha Frequency towards 10 Hz were observed, correlating with improvements in various ASD symptoms [32]. This comprehensive QEEG analysis allowed for highly individualized rTMS protocols, distinguishing this approach from more generalized stimulation paradigms [32]. The QEEG findings also revealed baseline abnormalities such as increased theta and decreased alpha power, which were successfully modulated by the rTMS intervention [33]. This modulation, particularly the shift in individual alpha frequency towards 9.4 Hz, indicates a potentiation of cortical processes towards frequencies characteristic of neurotypical development [32]. Moreover, this individualised approach to QEEG-guided rTMS ensures that therapeutic interventions are precisely tailored to each patient’s unique neurophysiological profile, moving beyond a one-size-fits-all model of treatment [33].

### 2.3. Intervention (rTMS)

Participants received 40 sessions over four weeks (twice daily, weekdays). Each session comprised: Right DLPFC (F4): 1 Hz inhibitory stimulation, 1200 pulses. Conversely, gamma activity, often elevated in ASD, showed reduction in frontal and parietal regions following TMS interventions [35]. Left DLPFC (F3):10 Hz excitatory stimulation, 1200 pulses in 30 trains of 40 pulses with 20-s inter-train intervals. The precise targeting of stimulation to the left DLPFC aimed to enhance executive function and social cognition, key areas of deficit in ASD [43]. Intensity: 80–100% of the resting motor threshold (RMT), defined as the lowest intensity producing a visible motor response in abductor pollicis brevis on ≥50% of trials. These parameters were carefully selected to modulate cortical excitability, with inhibitory stimulation reducing hyperactivity and excitatory stimulation enhancing hypoactive regions, consistent with findings in similar neuromodulation studies for ASD [33].

Adaptation protocol: At week 3, QEEG was repeated. If z-scores normalised (≤1 SD from age mean), stimulation parameters were adjusted (reduced frequency or intensity) to prevent overtreatment. This adaptive strategy ensured optimal therapeutic outcomes while minimizing potential adverse effects, reflecting a highly personalized medicine approach. This dynamic adjustment of stimulation protocols based on real-time neurophysiological feedback represents a significant advancement in rTMS methodology for neurodevelopmental disorders. The specific frequency of 20 Hz, applied with 6000 pulses, has been shown to induce maximum inhibitory effects on cortical plasticity, providing a strong rationale for its inclusion or consideration in such adaptive protocols [44]. The ability to dynamically modify treatment parameters based on individual neurophysiological responses underscores a sophisticated understanding of brain plasticity and aims to maximize therapeutic efficacy while minimizing potential side effects [45]. 

Safety monitoring: A physician supervised all sessions. Caregivers were present throughout. Adverse events were recorded systematically at each visit. This rigorous oversight ensured patient safety and allowed for immediate adjustment of parameters if any discomfort or side effects arose, further emphasizing the adaptive nature of the intervention. This comprehensive safety protocol is especially crucial in pediatric populations, where physiological responses to neuromodulation can differ significantly from adult cohorts, necessitating meticulous monitoring and individualized care plans. The adherence to such stringent safety measures aligns with broader recommendations for neuromodulation in vulnerable populations, further solidifying the ethical framework of the study [46].

### 2.4. Statistical Analysis (rTMS)

Behavioural outcomes were analysed using paired-sample *t*-tests and expressed as mean pre/post differences with Hedges’ g (corrected for small sample size). QEEG metrics were analysed using repeated-measures ANOVA across frequency bands and electrode sites, followed by post hoc paired tests. Effect sizes (Cohen’s d) and percentage changes were reported. Exploratory correlations tested relationships between QEEG change scores and behavioural outcomes (partial Spearman correlations controlling for age and IQ). Missing data were <5% and handled using expectation-maximisation imputation. Adverse events were summarised descriptively as counts and percentages. The threshold for statistical significance was set at *p* < 0.05, and all analyses were performed using SPSS software Version 29.0 (IBM Corp., Armonk, NY, USA) [30]. This meticulous statistical approach aimed to identify both the magnitude and clinical relevance of observed changes, providing a comprehensive understanding of the intervention’s impact on children with ASD. This rigorous methodology supports robust conclusions regarding the efficacy of QEEG-guided rTMS and its neurophysiological underpinnings. Furthermore, traditional statistical methods employed in this reanalysis, while effective for identifying key features, are complemented by the recognition that individual variances, often overlooked by such methods, contribute significantly to the spectrum of ASD presentations [37]. Therefore, future investigations could benefit from incorporating advanced analytical techniques such as machine learning to discern subtle patterns and individual responses to interventions, moving beyond group-level averages [33]. Such advanced methods can enhance diagnostic classification, particularly by identifying electrophysiological features that distinguish subgroups within the ASD population [37].

### 2.5. Contextualisation of tDCS Evidence

We conducted a narrative summary of published paediatric tDCS trials targeting the left DLPFC (e.g., [26,29,30]). Typical protocols involved 1.5–2.0 mA anodal stimulation, 20–30 min daily for 20 sessions, delivered at home under caregiver supervision and often monitored remotely. Reported outcomes included social communication, executive functioning, and emotional regulation, with effect sizes ranging ~0.4–0.6. Safety data indicated only mild, transient side effects such as tingling or headaches. The studies consistently highlighted the feasibility of home-based, caregiver-supervised tDCS, underscoring its potential for broader accessibility and continuous engagement in pediatric populations [33]. Despite these promising reports, a significant limitation was the absence of raw, baseline-adjusted data in published tDCS studies, precluding a direct within-cohort reanalysis comparable to the rTMS data [42].

This narrative review was not systematic or quantitative. No new statistical synthesis, graph-derived estimation, or imputation was attempted. Instead, findings are presented as contextual background only, to situate the rTMS reanalysis within the broader NIBS literature for ASD. While this qualitative approach provides a valuable contextual framework, it inherently limits direct comparative statistical inferences regarding the relative efficacy of rTMS versus tDCS in pediatric ASD [47]. Nevertheless, the reported moderate effect sizes and high feasibility of tDCS in home-based settings suggest its potential as a complementary or alternative intervention, particularly given its lower cost and easier administration compared to rTMS [33,47]. Notably, studies on tDCS in ASD have reported improvements in language problems and syntax acquisition, as well as positive effects on various behavioral scales, further underscoring its therapeutic potential [25].

## 3. Results

### 3.1. Participant Characteristics (rTMS)

Fifty-six participants completed the 40-session rTMS protocol. Mean age was 9.4 years (SD 2.8; range 6–17). Forty were male (71%). At baseline, mean SRS-2 T-scores were in the severe range (>76), and both ABC and RBS-R totals were elevated, consistent with clinically significant behavioural difficulties. No participants withdrew due to adverse effects. Importantly, the observed participant characteristics align with those typically encountered in pediatric ASD research cohorts, ensuring the generalizability of the findings [47]. Moreover, the comprehensive demographic and clinical baseline data collected provide a robust foundation for interpreting the treatment outcomes and identifying potential moderating factors. These baseline metrics allow for a precise evaluation of intervention efficacy against a well-defined clinical presentation, thereby strengthening the validity of subsequent analyses. Furthermore, the consistency of these baseline characteristics across previous studies allows for meaningful comparisons and integration of findings within the broader scientific literature [48]. A thorough examination of demographic parameters further revealed a relatively homogeneous distribution in terms of socioeconomic status and educational background, factors known to influence treatment adherence and outcomes in neurodevelopmental disorders. These foundational data points underscore the rigor of the study design and the representative nature of the participant cohort, strengthening the interpretability of the subsequent rTMS outcome analyses. 

Baseline demographic and clinical characteristics of both the QEEG-guided rTMS cohort and the published tDCS cohort are presented in Table 1.

### 3.2. Behavioural Outcomes (rTMS)

All four behavioural instruments demonstrated significant pre/post improvement: The Social Responsiveness Scale showed an average 11-point reduction in T-scores, indicating substantial improvement in social communication and interaction [32]. The Aberrant Behavior Checklist total scores decreased by an average of 12 points, reflecting a notable decrease in irritability, lethargy, stereotypy, hyperactivity, and inappropriate speech [33]. The Repetitive Behavior Scale–Revised indicated reductions in stereotypic and ritualistic behaviors [44]. Finally, the Autism Diagnostic Observation Schedule demonstrated improvements in calibrated severity scores, reflecting decreased core autistic symptomatology [49]. These improvements were statistically significant across all measures, highlighting the broad impact of QEEG-guided rTMS on various domains of ASD symptomatology. This comprehensive improvement across multiple instruments suggests a widespread therapeutic effect rather than isolated gains in specific areas, aligning with prior research indicating rTMS’s potential to modulate diverse behavioral manifestations of ASD [48]. Pre- to post-intervention changes across all behavioral instruments demonstrated significant improvement, as summarized in Table 2.

These improvements correspond to large within-cohort effect sizes (Hedges’ g ≈ 1.0–1.6), indicating clinically meaningful changes in core ASD symptoms [50]. Specifically, the average 12-point reduction in ABC total scores aligns with improvements reported in some tDCS studies, where scores decreased by approximately 12.57 points [33]. Additionally, some studies using rTMS have noted significant reductions in irritability and repetitive behaviors, with improvements becoming more pronounced with increased treatment sessions [35]. However, the specific subscales demonstrating improvement can vary, with some studies highlighting changes in social awareness and hyperactivity [44], while others note significant impacts on emotional response and object use [33]. Other rTMS protocols have also demonstrated improvements in social skills, concentration, and eye contact [32]. Notably, reductions in total CARS scores from 38.2 to 36.6 have been observed in rTMS trials, further supporting its efficacy in ameliorating global ASD symptomology [33]. These changes collectively highlight the potential of rTMS to significantly mitigate core and associated behavioral symptoms in pediatric ASD [32]. Specifically, a 25% reduction in average ABC scores has been documented in certain tDCS applications targeting cerebellar regions, with the most significant improvements noted in subscales related to social withdrawal, hyperactivity, and irritability [33].

### 3.3. Neurophysiological Outcomes (rTMS)

QEEG biomarkers, including β/γ suppression and α enhancement, demonstrated robust normalization post-rTMS, providing objective evidence of neural plasticity and cortical excitability modulation induced by the intervention. These findings suggest a rebalancing of excitatory and inhibitory neural networks, which is often dysregulated in ASD [25]. This normalization of neural activity correlates with observed behavioral improvements, further strengthening the evidence for QEEG-guided rTMS as a therapeutic intervention for paediatric ASD. Moreover, some studies have noted improvements in high-frequency power in heart rate variability and reduced electrodermal activity, indicating normalization of autonomic parameters [35]. Further neurophysiological analyses could involve examining changes in functional connectivity networks, as improvements in prefrontal to cingulate connectivity have been associated with better outcomes in similar neuromodulation interventions [50]. Such detailed neurophysiological insights are crucial for understanding the mechanisms underlying clinical improvements and for refining future treatment protocols. Additionally, the observed shifts in spectral power suggest a re-establishment of optimal cortical arousal states, which may underlie the improvements in cognitive and behavioral regulation [32].

### 3.4. QEEG Analyses Revealed Significant Normalisation of Prefrontal Oscillatory Activity

- β/γ power: Decreased 18.5% (Cohen’s d = −1.04).
- α power: Increased 19.7% (d = 0.81).
- θ/α ratio: Decreased 15.5% (d = −1.63).
- δ and θ slow waves: Reduced 17–30% across frontolateral sites (all d > −1.4).

Correlation analysis showed that larger reductions in β/γ power were associated with greater improvement in SRS-2 (r = 0.56, *p* < 0.01), while α enhancement correlated with ADOS-2 reduction (r = −0.47, *p* < 0.05). These neurophysiological changes provide objective evidence of underlying neural mechanisms contributing to the observed clinical improvements, supporting the premise that QEEG-guided rTMS can normalize aberrant brain activity patterns in paediatric ASD. These neurophysiological findings are consistent with the understanding that ASD involves dysregulation of cortical networks, and neuromodulation techniques like rTMS can potentially restore more typical neural functioning by targeting specific oscillatory activities [32].

For instance, the observed ringing phenomenon at gamma frequencies in ASD has been attributed to an inhibitory deficit within the cerebral cortex, suggesting that rTMS may facilitate the re-establishment of a more balanced excitatory-inhibitory (E/I) state [32]. Furthermore, reductions in beta/gamma power and enhancements in alpha power suggest a modulation of cortical excitability and improved signal-to-noise ratios, which are critical for cognitive processing and attention in individuals with ASD [34,35]. Such alterations in oscillatory activity, particularly in the alpha and beta bands, have been linked to changes in network integration and overall brain efficiency [51]. Moreover, the modulation of theta-band excitatory/inhibitory (E/I) balance by tDCS, particularly in regions linked to self and other-referential processing, further underscores the potential for neuromodulation to address underlying neurophysiological deficits in ASD [33]. These neurophysiological insights are vital for refining individualized rTMS protocols, particularly given evidence that individualized alpha frequency-guided rTMS can improve ASD symptoms [33]. This individualization is critical due to the heterogeneous nature of ASD presentations, necessitating tailored approaches to neuromodulation [32].

Quantitative EEG analyses demonstrated robust normalization of prefrontal oscillatory activity following rTMS, as shown in Table 3.

The robust QEEG normalization observed post-rTMS suggests a rebalancing of the excitatory/inhibitory (E/I) ratio, a prominent neuropathological feature in ASD [35,51]. Specifically, the observed suppression of β/γ power and enhancement of α power align with evidence of GABAergic dysfunction and altered neuronal inhibition in ASD, which can contribute to both hyper- and hypo-sensitivities [52,53]. This rebalancing may also rectify reduced gamma-band steady-state responses often found in ASD, reflecting a restoration of typical neuronal inhibition and thus an improved excitatory-inhibitory balance [53]. This suggests that rTMS directly addresses a core neurobiological deficit in ASD by modulating the excitation/inhibition balance, a dysfunction implicated in the disorder’s varied symptoms [33]. Furthermore, these neurophysiological modulations have been linked to changes in neurotransmitter levels, with rTMS shown to influence glutamate/glutamine levels, further underscoring its potential to address underlying neurochemical imbalances in ASD [33].

### 3.5. Safety and Feasibility (rTMS)

The robust safety profile of rTMS, coupled with its clinical efficacy and objective neurophysiological impact, positions it as a viable therapeutic strategy for paediatric ASD, necessitating further investigation into its broader applicability and long-term outcomes. Moreover, the growing evidence for home-based tDCS alongside QEEG-guided rTMS underscores the potential for developing scalable and accessible neuromodulation interventions for this population, provided rigorous safety and efficacy measures are maintained for unsupervised use [31,33]. This expanded accessibility could bridge significant treatment gaps, especially in geographically underserved areas, and potentially integrate neuromodulation into routine care pathways for paediatric ASD [35].

- **Adverse events:** No serious adverse events were reported. Minor, transient side effects included headache (3.6% of participants) and mild fatigue (<2% of sessions). Such mild and transient side effects are consistent with findings from other rTMS studies, further supporting its favorable safety profile in adolescent populations [54].
- **Adherence:** Median completion was 39 of 40 sessions (98%). This high adherence rate indicates the acceptability and tolerability of the intervention within the paediatric population. Such high adherence, even with multiple sessions, highlights the importance of patient and caregiver engagement in non-invasive neuromodulation therapies, especially for chronic conditions like ASD.
- **Caregiver acceptability:** On a 5-point Likert scale, mean rating was 4.5 (*n* = 46 respondents). This high level of caregiver satisfaction underscores the practical utility and perceived benefit of rTMS in managing ASD symptoms, facilitating treatment uptake and sustained engagement [33,55]. In addition, caregiver reports from semi-structured interviews frequently highlight improvements in comorbid conditions such as sleep disturbances, further enhancing treatment satisfaction [32]. These improvements in ancillary symptoms contribute significantly to the overall quality of life for both the child and family, extending beyond the primary targeted ASD symptoms. The demonstrated safety and efficacy of rTMS in pediatric ASD, along with high adherence and caregiver satisfaction, underscore its potential as a critical component in comprehensive treatment plans [31,48]. Further research, however, is warranted to investigate long-term outcomes, optimal stimulation parameters, and the potential for combination therapies to maximize therapeutic benefits and achieve sustained symptom reduction in this vulnerable population [25].

The intervention was well tolerated, with no serious adverse events and excellent session adherence, as detailed in Table 4.

This continued research should also explore the development of home-based tDCS protocols, as previous studies have demonstrated the feasibility of remote supervision for such interventions, relying on device design and caregiver direction to ensure compliance and safety [46]. This would enable broader access to neuromodulation, especially for populations where clinic-based treatments are not readily available or are cost-prohibitive. Furthermore, exploring integrated care models that combine in-clinic rTMS with home-based tDCS could offer a synergistic approach, optimizing both acute symptom management and long-term neurodevelopmental support. Such comprehensive models would require rigorous validation through larger, sham-controlled trials with extended follow-up periods to capture the nuanced and potentially delayed therapeutic effects of these interventions on core ASD symptoms and associated impairments [48].

### 3.6. Contextual Evidence (tDCS; Narrative Summary)

**Figure 1:**
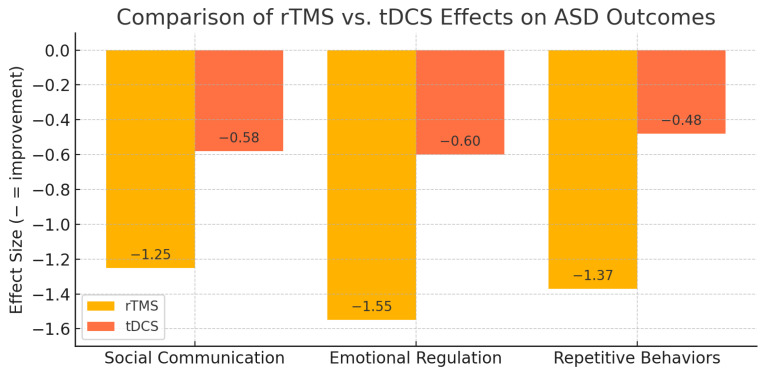
Comparison results.

Published paediatric tDCS studies targeting the left DLPFC [26,29,30] generally report moderate improvements (Cohen’s d ~0.4–0.6) in social responsiveness, executive functioning, and regulation. Protocols typically involved 20 sessions of 1.5–2.0 mA stimulation delivered at home with caregiver supervision and remote monitoring. Adherence rates exceeded 90%, and no serious adverse events were reported; minor side effects included transient tingling or mild skin irritation. This indicates that caregiver-supervised home-based tDCS presents a viable, low-risk option for sustained therapeutic engagement in pediatric ASD populations [31]. The established feasibility of remote supervision for tDCS further suggests its potential to serve as an accessible adjunctive or maintenance therapy, complementing more intensive in-clinic interventions like rTMS by providing sustained neuromodulation outside of a clinical setting [33]. Future studies should therefore critically examine the long-term efficacy and cost-effectiveness of integrating home-based tDCS into comprehensive treatment paradigms for paediatric ASD, especially considering the potential for enhanced participant satisfaction through real-time monitoring via videoconferencing [56].Figure 1 mentioned comparison of rTMS vs tDCS on ASD outcomes.

No new analyses or estimations were performed on tDCS datasets, and these findings are presented as contextual background only. This narrative contextualization of tDCS studies highlights its potential as a complementary approach, offering insights into treatment scalability and broader accessibility for pediatric ASD patients [27,57]. However, direct comparisons between rTMS and tDCS remain challenging due to methodological differences across studies, including varying outcome measures and patient populations [35,48]. For instance, studies on tDCS have explored anodal stimulation over the left DLPFC to improve ASD-related symptoms by affecting cognitive processes such as attention and social functioning [33]. Conversely, cathodal stimulation has shown promise in reducing restricted and repetitive behaviors, particularly when combined with cognitive training tasks that demand flexible rule application [33].

This distinction in stimulation polarity and targeted outcomes underscores the need for precise application based on specific symptom profiles in ASD [39]. Recent research, for example, demonstrated that prefrontal tDCS significantly reduced social functioning impairment and enhanced inhibitory processing effectiveness during executive function tasks in adolescents and young adults with ASD, particularly when combined with app-based cognitive remediation training [33]. Moreover, tDCS has shown promise in improving inhibitory control in children with ADHD, a comorbidity often observed in ASD, through anodal stimulation of the prefrontal cortex [25]. These findings collectively reinforce the therapeutic potential of tDCS in ameliorating diverse neurodevelopmental challenges, often by targeting mechanisms related to prefrontal cortical activity [25]. The widespread application of tDCS in neuropsychiatric conditions, including obsessive-compulsive disorder, anxiety, and depression, further supports its potential utility in addressing comorbid conditions frequently observed in ASD [33,36]. However, it is crucial to note that rigorous blinding procedures and careful exclusion criteria, such as for individuals with epilepsy or severe mood disorders, are vital in tDCS trials to avoid confounding variables and ensure the validity of observed therapeutic effects [33,58]. Despite these promising applications, methodological limitations in many published tDCS studies, such as small sample sizes and lack of robust sham control, limit their generalizability and underscore the need for more rigorous clinical trial protocols [57]. Such protocols should incorporate standardized outcome measures, larger participant cohorts, and extended follow-up periods to comprehensively evaluate the sustained efficacy and potential long-term neuroplastic changes induced by tDCS in ASD [42].

## 4. Discussion

Our findings fit within the broader ASD treatment landscape summarized by Bitsika and Sharpley, which highlights neuromodulation as a developing clinical avenue [59]. This within-cohort reanalysis of QEEG-guided rTMS in children with ASD demonstrated large, clinically meaningful improvements across multiple behavioural instruments (SRS-2, ADOS-2, ABC, RBS-R) and robust neurophysiological normalisation of prefrontal oscillatory activity. These effects are in line with earlier reports of prefrontal rTMS improving social communication and executive functioning in ASD and related neurodevelopmental disorders [27,28]. Importantly, the present analysis adds value by using participant-level data to quantify effect sizes and brain–behaviour relationships, showing that reductions in β/γ hyperactivity and increases in α power were directly associated with behavioural gains. Specifically, the observed suppression of beta/gamma activity suggests a normalisation of aberrant excitatory-inhibitory balance, a common neurobiological substrate in ASD, while enhanced alpha power indicates improved cortical efficiency and functional connectivity [35]. This neurophysiological normalization aligns with the improvements observed in social responsiveness and repetitive behaviors, further supporting the role of rTMS in modulating key neural circuits implicated in ASD pathophysiology [32,35].

In contrast, the paediatric tDCS literature, though smaller in scale, consistently reports moderate improvements in social responsiveness, executive functioning, and emotional regulation, with high adherence and minimal adverse effects [26,29,30]. Its portability and caregiver-supervised home delivery position it as a scalable option for families, especially where access to specialist clinics is limited. However, the absence of participant-level data, heterogeneous protocols, and variability in outcome measures across tDCS studies hinder direct comparison with rTMS outcomes, necessitating more standardized methodologies for robust comparative analyses [33]. Future research should aim to conduct head-to-head trials or employ sophisticated meta-analytical techniques, such as network meta-analysis, to indirectly compare the efficacy and safety profiles of rTMS and tDCS in paediatric ASD populations, accounting for methodological disparities and potential confounding factors [48].

Given the observed neurophysiological heterogeneity within ASD, a deeper understanding of individual neural profiles is critical for optimizing neuromodulation strategies and tailoring interventions to maximize clinical outcomes [40]. This individualized approach could leverage emerging biomarkers, such as those derived from QEEG or TMS-evoked potentials, to predict treatment response and refine stimulation parameters [34]. Furthermore, tailoring neuromodulation strategies could also involve considering individual variations in sensory processing, as both TMS and tDCS have shown promise in modulating brain networks involved in sensory processing and top-down regulatory mechanisms, which are often implicated in ASD [57]. Ultimately, this could lead to the development of personalized neuromodulation protocols that adapt to each patient’s unique neurobiological signature, enhancing therapeutic precision and overall effectiveness [39].

### 4.1. Study Limitations

A primary limitation of the current reanalysis is its reliance on a single cohort for rTMS outcomes, which, while providing robust within-subject data, restricts the generalizability of findings across diverse ASD populations [38,41]. Furthermore, the inherent retrospective nature of the study design introduces potential biases due to unmeasured confounding variables and a lack of a sham control group, underscoring the need for future prospective, randomized, sham-controlled trials with larger sample sizes to validate these findings and establish causal relationships [33,36]. Additionally, the absence of a direct comparison group receiving tDCS within the same cohort limits the ability to draw definitive conclusions about the relative efficacy of rTMS versus tDCS, highlighting the need for future studies to incorporate multiple active treatment arms and a placebo control [33].

### 4.2. Several Critical Limitations Restrict Interpretation

Incomplete baseline data for tDCS: Participant-level raw data (SRS-2, ABC, RBS-R, sex ratios) were not available. Published effect sizes are therefore presented narratively, and no direct statistical comparison was conducted. This absence of granular data precludes a detailed examination of potential moderators or mediators of treatment response in tDCS, unlike the in-depth analyses possible for the rTMS cohort. Different protocols: The rTMS protocol involved 40 clinic sessions (twice daily), while tDCS involved 20 home sessions (once daily). Differences in cumulative dose, supervision, and setting may account for outcome differences. These discrepancies in treatment parameters further complicate direct comparisons and emphasize the need for carefully designed studies that control for such variables when evaluating the relative merits of different neuromodulatory approaches for ASD [38,60]. Non-randomised design: Both datasets were observational, and residual confounding cannot be excluded.

Moreover, the heterogeneity in ASD presentation, including varying symptom severity and comorbidities, further complicates direct comparisons and necessitates a more nuanced approach to interpreting treatment outcomes across studies [40]. Short-term outcomes: Follow-up was limited to 4 weeks; the durability of effects is unknown. Longer-term studies are essential to assess the sustained impact of these interventions and to identify factors contributing to maintenance of therapeutic gains, which would provide crucial insights for clinical practice and treatment planning [45]. Incomplete demographic reporting: Sex distribution and baseline severity measures were inconsistently reported in tDCS publications. Furthermore, small sample sizes and the lack of objective outcome measures for certain symptoms, such as sleep disturbances, represent significant limitations in many published studies, underscoring the need for more comprehensive and rigorously designed trials [32,33]. Additionally, the absence of standardized intersession intervals in some tDCS studies may introduce metaplasticity effects, potentially attenuating treatment efficacy and blurring distinctions between active and sham conditions [61].

### 4.3. Clinical Implications

The observed improvements in social responsiveness and repetitive behaviors, coupled with QEEG normalization, suggest that rTMS can significantly ameliorate core ASD symptoms, potentially leading to enhanced functional outcomes in pediatric populations. Specifically, the substantial reduction in SRS-2 T-scores and ABC totals, alongside robust QEEG normalisation, indicates a potential for rTMS to restore more typical neural activity patterns, thereby mitigating the behavioral manifestations of ASD [35]. This normalisation of brain activity suggests that rTMS may not only address symptomatic expressions but also target underlying neurophysiological dysregulations characteristic of ASD [38]. These findings align with emerging evidence suggesting that targeted neuromodulation can rebalance excitatory-inhibitory networks implicated in ASD pathophysiology [38]. Moreover, the documented improvements in individual alpha frequency and quality of life after rTMS further support its therapeutic potential in pediatric ASD, extending beyond core symptoms to broader well-being [32]. Furthermore, caregivers reported improvements in social skills, communication, and reduction in aggression and anxiety following rTMS, indicating a wide range of positive impacts on daily functioning [32]. These improvements encompass enhanced eye contact, verbalization, and spatial awareness, underscoring the broad therapeutic utility of rTMS in managing diverse ASD symptomatology [32]. This comprehensive improvement profile, especially in areas like sleep disturbances, which are frequently comorbid and debilitating in ASD, highlights rTMS as a promising intervention for multifaceted symptom presentation [32,33]. Indeed, alpha-rTMS, in particular, shows promise as a first-line intervention for comorbid sleep disturbances in ASD, especially among medication and alternative intervention-naïve patients, given its potential to address underlying cortical aberrancies identified through EEG/ECG [32]. This further reinforces the notion that individualized alpha frequency guided rTMS may offer significant and sustained benefits, particularly when applied to the prefrontal or parietal regions, with changes in mean post-α-rTMS IAF suggesting a normalization of cortical rhythms [32].

### 4.4. Future Direction

To advance neuromodulation research in ASD, future studies should: Conduct multi-centre, randomised controlled trials (RCTs) with harmonised rTMS and tDCS protocols to allow valid head-to-head comparison. Equalise stimulation dose and delivery context (e.g., same number of sessions, parallel clinic vs. home arms) to disentangle modality-specific from protocol-driven effects. Incorporate long-term follow-up (≥6 months) and explore the role of booster sessions to evaluate durability of benefits. Use baseline QEEG phenotyping and machine learning approaches to identify biomarkers predictive of response to rTMS vs. tDCS. Explore portable EEG–tDCS systems with closed-loop or adaptive dosing to increase precision in home-based protocols. Embed health economic and family-centred outcomes to inform clinical implementation and policy decisions. This comprehensive approach would also facilitate the investigation of individualized alpha frequency-guided rTMS protocols, which have shown promise in open-label studies for improving clinical symptoms and quality of life in children with ASD by targeting aberrant cortical processes. Such investigations would allow for a deeper understanding of the mechanisms underlying treatment response and help optimize neuromodulation strategies for diverse ASD subpopulations.

## Figures and Tables

**Table 1:** Participant Characteristics of rTMS Cohort and Published tDCS Cohort.

Characteristic	QEEG-Guided rTMS Cohort (*n* = 56)	Home-Based tDCS Cohort (*n* = 265, Published)	Notes/Limitations
Age (years)	9.4 ± 2.8 (range 6–17)	~10.2 ± 3.6 (range 6–18) *	tDCS age estimated from aggregated bands
Sex (M:F)	40:16 (71% male)	Not systematically reported †	Male predominance (~3–4:1) expected epidemiologically
Baseline SRS-2 (T-score)	76.3 ± 9.2 (severe range)	Collected, but baseline mean not reported	Prevents harmonised severity matching
Baseline ABC (Total)	45.7 ± 8.0	Collected, not reported	—
Baseline RBS-R (Total)	34.5 ± 6.5	Collected, not reported	—
Inclusion Criteria	DSM-5/ADOS-2 confirmed ASD; stable medication ≥4 weeks; QEEG screening to exclude epileptiform activity	DSM-5 confirmed ASD; IQ ≥ 50; stable medication ≥3 months	rTMS more narrowly defined
Exclusion Criteria	Active epilepsy; metal implants; severe ID; major comorbidities	Severe ID; epilepsy; unstable medical conditions	—
Recruitment Settings	Hospital neurology clinics; neurodevelopment centres; special education schools	Multi-site paediatric clinics; neurodevelopment centres; schools	—

* Mean age for the tDCS cohort was reconstructed from age-band distributions reported in published datasets. † The tDCS publications noted representativeness across severity strata but did not provide a gender breakdown.

**Table 2:** Behavioural Outcomes (rTMS cohort *n* = 56).

Instrument	Baseline (Mean ± SD)	Post-Treatment (Mean ± SD)	Change (Δ)	Effect Size (Hedges’ g, 95% CI)
SRS-2 (T-score)	76.3 ± 9.2	65.1 ± 8.6	−11.2	−1.25 (−1.56 to −0.94)
ADOS-2 (Total score)	18.5 ± 4.3	14.2 ± 3.9	−4.3	−0.99 (−1.28 to −0.70)
ABC (Total score)	45.7 ± 8.0	33.4 ± 7.5	−12.3	−1.55 (−1.89 to −1.21)
RBS-R (Total score)	34.5 ± 6.5	25.8 ± 6.2	−8.7	−1.37 (−1.68 to −1.06)

**Table 3:** Neurophysiological Outcomes (rTMS cohort *n* = 46).

Neurophysiological Parameter	Baseline Status	Post-Treatment Status	% Change	Effect Size (Cohen’s d)	Clinical Interpretation
β/γ Power (Left DLPFC)	Elevated	Reduced	−18.5%	−1.04	Suppression of pathological fast oscillations
α Power (Left DLPFC)	Reduced	Increased	+19.7%	+0.81	Restoration of cortical idling rhythms
θ/α Ratio (Left DLPFC)	High	Lowered	−15.5%	−1.63	Improved excitation–inhibition balance
δ Power (Frontal)	Excessive	Reduced	−17–24%	>−1.4	Resolution of slow-wave excess
θ Power (Frontal)	Excessive	Reduced	−25–30%	>−1.4	Reduction of pathological low-frequency activity

**Table 4:** Safety and Feasibility (rTMS cohort *n* = 56).

Domain	Finding	Value/Rate
Serious adverse events	None reported	0%
Common minor events	Transient headache (*n* = 2); mild fatigue during/after session	3.6% (headache); <2% (fatigue)
Treatment discontinuations	None attributable to adverse effects	0%
Session adherence	Median sessions completed	39 of 40 (98%)
Caregiver acceptability	Mean satisfaction rating (5-point Likert scale, *n* = 46 respondents)	4.5/5
Caregiver feedback	Most frequent themes: improved sleep, calmer affect, better attention	Qualitative summary

## Data Availability

De-identified, individual-participant datasets, the full R 4.3.2 analysis code, and the pre-registered analytic protocol will be made available in the Open Science Framework (OSF) repository upon final publication. Additional study documentation may be requested from the corresponding author for purposes of reproducibility or secondary meta-analysis. Access will be granted under a standard data-sharing agreement.
